# Antitumor activities of novel glycyrrhetinic acid-modified curcumin-loaded cationic liposomes *in vitro* and in H22 tumor-bearing mice

**DOI:** 10.1080/10717544.2018.1526227

**Published:** 2018-11-30

**Authors:** Mingxiang Chang, Meimei Wu, Hanmin Li

**Affiliations:** a First clinical medical school, Hubei University of Chinese Medicine, Wuhan, P.R. China;; b Hubei Provincial Hospital of Traditional Chinese Medicine, Wuhan, P.R. China;; c Hubei Province Academy of Traditional Chinese Medicine, Wuhan, P.R.China

**Keywords:** Curcumin, glycyrrhetinic acid, glycyrrhetinic acid modified curcumin-loaded cationic liposomes, H22 hepatoma transplanted tumor, antitumor effects

## Abstract

At present, the chemotherapy of advanced inoperable liver cancer is limited with serious side effects. Curcumin possesses multiple cancer preventive activities and low safety concerns. However, its poor solubility and instability in water pose significant pharmacological barriers to its clinical application. In this study, we presented a novel delivery system – the glycyrrhetinic acid modified curcumin-loaded cationic liposomes (GAMCLCL) and investigated its antitumor activities on HepG2 cells *in vitro* and in H22 tumor-bearing mice. The experimental results demonstrated that GAMCLCL was a cationic liposome and could be Intravenous administration. Compared to free curcumin, GAMCLCL exhibited stronger antitumor activities *in vitro* and *in vivo*. The antitumor results of GAMCLCL after intravenous administration were very similar to those after intratumoral administration. The main activities of GAMCLCL and curcumin included inhibition of HepG2 cell proliferation, inhibition of tumor growth, reduction of tumor microvascular density, down-regulation of the expression of VEGF protein, and up-regulation of the expression of Caspases3 protein in H22 tumor tissues. Furthermore, GAMCLCL improved the parameters of WBC, RBC, ALT, CRE, LDH of H22 tumor-bearing mice. Curcumin is a nontoxic natural compound with definite antitumor activities, its antitumor effects can be enhanced by preparation of GAMCLCL.

## Introduction

Hepatocellular carcinoma (HCC) is one of the most common human malignancies worldwide with high morbidity (Liao et al., 2014). The global incidence of HCC has doubled over the last two decades, particularly in the developing countries (Bender, 2014; Ferlay et al., 2015). In patients with advanced inoperable liver cancer, chemotherapeutic agents are the predominant treatment; however, the effects are usually unsatisfactory and serious side effects may occur owing to poor selectivity to cancerous cells (Wysocki, 2010; Forner et al., 2012; Lin et al., 2012).

Most of the conventional chemotherapeutic agents used today were designed to hit a single intracellular target. As cancer is a multifactorial disease, the treatment may require compounds able to target multiple intracellular components. Therefore, exploration of compounds with fewer and weaker side effects, in addition to the capability to affect multiple targets, is urgently required. Recently, natural medicines with improved effectiveness and lower toxicity have received more and more attention as a potential source of new therapeutic antitumor drugs for patients with HCC (Wang et al., 2014; Wu et al., 2014).

Curcumin and Glycyrrhetinic acid (GA) are the main active ingredients of Diwuyanggan capsule (a hospital preparation, prepared by our research group). A series of experimental and clinical studies have shown that the capsule exerts anti-HCC, anti-liver injury, anti-hepatic fibrosis, inhibits HBV replication, regulates immunity and modulates liver regeneration, and reduces the liver cancer risk in HBeAg-negative patients with chronic hepatitis B (Hanmin et al., 2014; Xin et al., 2014; Hanmin, 2015; Hanmin et al., 2015; Zhao et al., 2015; Hanmin & Zhang, 2017).

Curcumin, a hydrophobic polyphenolic compound extracted from the rhizomes of *Curcuma longa,* has been used in curry and as a traditional medicine for many centuries in Ayurvedic, Chinese, and Hindu medicine systems. It was demonstrated to be safe at doses up to 12 g/day in clinical studies (Cheng et al., 2001; Sharma et al., 2001; Chainani-Wu, 2003; Sharma et al., 2004; Lao, 2006). Curcumin, granted ‘Generally Recognized as Safe’ by the Food and Drug Administration (FDA), exhibits various biological activities, including anti-inflammatory, anti-atherosclerotic, anti-proliferative, anti-oxidant, anti-cancer, and antimetastatic effects. Efficacy as a cancer preventive compound has also been demonstrated in experimental systems of liver cancer, breast cancer, gastric cancer, colorectal cancer, esophageal cancer, skin cancer, lymphoma, and leukemia (Maheshwari et al., 2006; Jurenka, 2009; Bar-Sela et al., 2010). The USA National Cancer Institute (NCI) has listed it as a third generation of cancer chemo-preventive drug (Churchill et al., 2000). However, curcumin is a poorly soluble, rapidly metabolized, with poor bioavailability, instability in water, and photo-instability, which limit its use as an effective therapeutic agent.

In an effort to address these limitations, in the previous study, we found that cationic liposomes could form complex with curcumin, which significantly improved the inhibitory effect of curcumin on the Bel-7402, and the water solution of the Lipoplexes could maintain the inhibitory effects on the Bel-7402 cells for 12 months (Hanmin et al., 2005).

Although cationic liposomes can improve the physical limitations of curcumin, their delivery to the specific target site remains a major challenge. To enhance the distribution of liposomes to target tissues, researchers usually modified the liposome surfaces with ligands or small molecules (Gayong et al., 2013).

Glycyrrhetinic acid has been used to target hepatocellular carcinoma cells, based on a study showing that protein kinase C, a binding target of glycyrrhetinic acid, is expressed more highly on the surface of hepatocellular carcinoma cells compared to adjacent non-tumor liver cells (O’Brian et al., 1990). It was also reported that liposomes modified with GA exhibited markedly high affinity for hepatocytes (Mao et al., 2005; Lin et al., 2008; Abu-Lila et al., 2009) and Significantly enhanced proliferation inhibition on tumor cells (Sayoko et al., 1994; He et al., 2010; Mingrong et al., 2013; Jingde et al., 2015; Shaomei et al., 2016). In addition, our previous research illustrated that the combination of curcumin and GA enhanced the inhibition of the proliferation and promoted the apoptosis of HepG2 cells (Mingxiang et al., 2017).

In this study, we synthesized a guide complex of GA and octadecylamine through a simple static binding reaction, which was used to prepare novel GA modified curcumin-loaded cationic liposomes (GAMCLCL). The antitumor effects of GAMCLCL and the potential mechanisms of action were analyzed *in vitro* and in H22 tumor-bearing mice.

## Materials and methods

### Chemical and reagents

Curcumin was purchased from Hangzhou sky grass technology company Ltd (Hangzhou, China). Glycyrrhetinic acid (GA) was from Hubei Yuanda Pharmaceutical industry (Wuhan, China). Complexes of GA and octadecylamine (CGO) were synthesized in our laboratory (Hubei University of Chinese Medicine, China) based on improvements on a previous report (Hui et al., 2006). Adriamycin was from Shenzhen Main Luck Pharmaceuticals Inc. (Shenzhen, China). Lecithin was from Shanghai AVT Technology Pharmaceutical Ltd (Shanghai, China). Octadecylamine was from Sigma Company (USA). RPMI-1640 culture medium and double resistance were from Hyclone Company (USA). Mycoplasma-free fetal bovine serum was from Sijiqing Technology Co., Ltd(China). Pancreatic enzymes were obtained from Gibco Company (USA). The Immuno – Bridge + kit was from GBI Company (USA). The LDH-kit was from Nanjing Jiancheng Bioengineering Institute (Nanjing, China). Anhydrous ethanol was from National Medicine Chemical Agent Co., Ltd (China).

### Cell line and animal

The HepG-2 cell (CL-0103) and H22 cell line (CL-0341) were purchased from Procell Company (Wuhan, China). Male kunming mice (weight 20 ± 2.0 g), were obtained from the Medical Animal Test Center of the Hubei Disease Control Center (Wuhan, China). All mice were maintained in a specific pathogen-free environment at the Animal Experiment Center of Hubei University of Chinese Medicine, and all procedures were approved by the Animal Care and Use Committee of University and conformed to the National Act on the Use of Experimental Animals (People’s Republic of China).

### Preparation of GAMCLCL

In a 50 °C water bath, CGO 25 mg, lecithin 200 mg, and curcumin 8 mg were dissolved in 3 mL of anhydrous ethanol and left to stand for 10 min. One milliliter of the residue solution was injected slowly into 20 ml of preheated (50 °C) and stirred (250 rpm) double distilled water. The solution was stirred (250 rpm) at 50 °C until the ethanol was completely evaporated, and kept at room temperature for 24 hours, and filtered through a 200 nm microporous membrane to obtain GAMCLCL, which was stored at 4 °C in a sealed container. A cationic liposome without curcumin, prepared by the same method, was used as a blank control.

### Characteristics of GAMCLCL

An appropriate amount of GAMCLCL was diluted 30 times with double distilled water. The morphology of GAMCLCL was evaluated by using a transmission electron microscope (Japan), and the particle size and potential were determined by using a ZS90 Nanoparticle instrument (Malvern, UK). Transparent single-use tubes were used to observe the aggregation and precipitation of a 1:3 mixture of GAMCLCL solution and fetal bovine serum after 0, 0.5, 1, 2, 4, 8, 24, 48, 72 hours, respectively.

### Entrapment efficiency of GAMCLCL

Entrapment efficiency (EE) and drug loading (DL) were determined by centrifugation at 70,000 rpm and 4.0 °C for 2 hours. The amount of curcumin in the supernatant and in 1 mL of liposomes was analyzed by high-performance liquid chromatography (HPLC, Waters alliance 2695, USA) at 420 nm. The EE and DL of GAMCLCL were calculated from Equation 1 and Equation 2, respectively:

(1) $$\text{EE}=(M_{\text{liposome}}\;-\;M_{\text{supernant}})/M_{\text{liposome}}\times 100\%$$

(2) $$\text{DL}=M_{\text{curcumin}}\times \text{EE}/M_{\text{lipids}}\times 100\%$$

M_curcumin_ is the total amount of curcumin in the sample; M_lipids_ is the total amount of lipids in the sample.

### Hemolysis assay

Prior to the hemolysis assay, fresh rat blood was collected and prepared as a 2% (v/v) RBC suspension. According to the standard operating method, the 2.5 mL of RBC suspension was added in 7 tubes, respectively; In No.1 ∼5 tubes, GAMCLCL (0.5, 0.4, 0.3, 0.2, 0.1 mL), and saline (2, 2.1, 2.2, 2.3, 2.4 mL) was added and shaken, respectively; In No.6, 2.5 mL of deionized water was added, which was used as the positive control; In No.7, 2.5 mL of saline was added, and used as the negative control. After incubation at 37 °C for 3 hours, the mixture was centrifuged at 3000 rpm for 10 min, the supernatant was collected and the hemoglobin release was analyzed by using an automatic enzyme standard instrument (Multiskan MK3, USA) at 545 nm. The hemolysis ratio (HR) was calculated as follows:

$$\text{HR}=(A_{\text{sample}}\;-\;A_{\text{negative}})/(A_{\text{positive}}\;-\;A_{\text{negative}})\times 100\%$$

A_sample_, A_negative_, and A_positive_ are the absorbance of the samples, negative control, and positive control, respectively.

### Vascular irritation study in rabbit

The vascular irritation of the GAMCLCL was evaluated in the ear marginal veins of rabbits. Six rabbits (2.0–2.5 kg) were randomly divided into two groups (GAMCLCL and control). The GAMCLCL group was injected with 20 mL GAMCLCL through the marginal veins of the right ears at a rate of 2 mL/min; the control group was injected 20 mL saline by using the same method. All injections were performed every day for 5 days. The rabbits were killed 2 days after the final injection and two parts of the ear vein were obtained for observation (the proximal region and the distal region from the pinprick). Histopathological examination was performed to evaluate the degree of vascular irritation.

### Cell proliferation inhibition assay

The inhibition of HepG-2 cell proliferation was assessed by CCK-8 Kit (Biosharp, Hefei, China). Cells (5000/well) were seeded in 96-well plates with 100 µL/well medium and incubated overnight with 10% fetal bovine serum 1640 medium at 37 °C and in an atmosphere of 5% CO_2_. The following treatments were applied: 20, 15, 10, 5, and 2.5 μg/mL curcumin; 20, 15, 10, 5, and 2.5 μg/mL GAMCLCL (containing equivalent curcumin concentration); the blank liposome group (dilution as for the GAMCLCL group); untreated cells were used as the control (with 100% cell viability), and the medium without cells was used as the blank. After treatment for 24, and 48 hours, respectively, the medium was removed and 1640 medium containing 10% CCK-8 was added. After incubation for 30 min at 37 °C, the absorbance (A) at 450 nm was measured by using an automatic enzyme standard instrument (Multiskan MK3, USA. The inhibition rate (IR) of cellular proliferation was calculated from the following equation:

$$\text{IR}=(1\;-\;A_{\text{experimental}}{}_{\text{group}})/A_{\text{control}\text{group}}\times 100\%$$

### Cellular uptake *in vitro*

HepG2 cells (5 × 10^5^cells/well) were seeded in 6-well plates with 1640 medium, after incubated overnight at 37 °C and in an atmosphere of 5% CO_2_, the medium was removed, the cells were washed twice with PBS, and the following treatments were applied: blank group, only 1640 medium was added; curcumin group, 1640 medium containing 10 μg/mL curcumin was added; GAMCLCL group, 1640 medium containing GAMCLCL equivalent to 10 μg/mL curcumin was added. After incubated for 1, 2, and 4 hours, respectively, the medium was removed, the cells were carefully washed twice with PBS, and the cells were digested with 0.25% trypsin without EDTA. After digestion, the solution was centrifuged (5 min at 1500 rpm) to harvest the HepG2 cells, which were re-suspended in 500 μL pre-cooled PBS. The uptake of curcumin was measured by flow cytometry (BD Biosciences, USA) at an excitation wavelength of 488 nm and an emission wavelength of 530 nm.

### Cellular apoptosis *in vitro*

HepG2 cells (5 × 10^5^cells/well) were seeded in 6-well plates with 1640 medium. After incubated overnight at 37 °C and in an atmosphere of 5% CO_2_, the medium was removed, the cells were washed twice with PBS, and the following treatments were applied: blank group, only 1640 medium was added; Curcumin group, 1640 medium containing 10 μg/mL curcumin was added; GAMCLCL group, 1640 medium containing GAMCLCL (equivalent to containing 10 μg/mL curcumin) was added. After incubation for 24 hours, the medium was removed, and the cells were carefully washed twice with PBS. After digestion with 0.25% trypsin without EDTA, the cells were centrifuged for 5 min at 1200 rpm and harvested. The AnnexinV-APC/7-AAD kit (KGA1026, Nanjing, China) was used for the flow cytometry (BD Biosciences, USA) evaluation of apoptosis.

### H22 hepatoma transplanted tumor model

Under sterile conditions, the mice were vaccinated using the H22 hepatoma cell line in enterocoelia for 7 days, the ascites were collected, diluted with sterile saline, and the cell concentration was adjusted to 1 × 10^7^/mL. The diluted solution (0.2 mL) was administered subcutaneously in the right fore axillary region of each mouse, who were then fed under normal conditions.

### Intratumoral injection in H22 tumor-bearing mice

The inoculated mice were randomly divided into 6 groups, and 6 mice per group. After 5 days of vaccination, there was a clear tumor mass in the right fore axillary subcutaneous region of the mice. Subsequently, the mice received an intratumoral injection every day for 7 days, as follows: the model group was injected saline at the same volume as the other group; the adriamycin group was injected with 1 mg/kg adriamycin; the curcumin group was injected 20 mg/kg curcumin diluted in ethanol; the high-, middle-, and low-dose GAMCLCL groups were injected 20, 10, and 5 mg/kg (equivalent to containing curcumin) of GAMCLCL solution, respectively; and 6 normal mice were set as the normal control group and injected with an equal volume of saline. During the experimental period, the routine actions of the mice were observed and recorded.

After the 7-day administration period, all mice were weighed on the eighth day and were sacrificed by cervical dislocation. In each mouse, the tumor was separated, and a Vernier caliper (Sega tooling, Shenzhen, China) was used to measure the tumor maximum diameter (a) and minimum diameter (b).

The tumor volume (V) was calculated from the following equation：

$$V=a\cdot b^{2}/2\left({\text{mm}}^{3}\right).$$

The tumor growth inhibition rate (IR) was calculated from the following equation:

IR=[(A−B)/A]×100%.

A is the average tumor weight of the model group; B is the tumor weight of the treated group.

The net body weight (NBW) of mice was calculated from the following equation:

NBW = the whole body weight of H22 bearing mouse–the tumor weight (g).

### Intravenous injection in H22 tumor-bearing mice

The inoculated mice were randomly divided into 6 groups, and 6 mice per group. After the vaccination for 3 days, the tumors that appeared in the right fore axillary subcutaneous region of mice were approximately the same size as a soya bean. Through daily injection into the tail vein for 7 days, the mice were then administered the following treatments: the model group were injected saline at the same volume as the other groups; the adriamycin group were injected 2 mg/kg adriamycin; the curcumin group were injected 20 mg/kg curcumin diluted in ethanol; the high-, middle-, and low-dose GAMCLCL groups were injected 20, 10, and 5 mg/kg (equivalent to containing curcumin) of GAMCLCL solution, respectively; and 6 normal mice were set as the normal control group and injected with an equal volume of saline. During the experiment, the routine actions of mice were observed and recorded.

After the blood was collected from the mice, the mice were sacrificed by cervical dislocation, and the tumors were separated from every mouse. The tumor volume (V), tumor growth inhibition rate(IR), the net body weight (NBW) were calculated by using the equations as above.

### Blood biochemical examination after IV administration

After the 7-day administration, each mouse was weighed on the eighth day, blood samples were collected, and the serum samples were harvested by using centrifugation. The red blood cell (RBC) count, white blood cell (WBC) count, hemoglobin (HGB), alanine aminotransferase (ALT) were measured in the blood samples, and creatinine (CRE) was evaluated in the serum samples by using automatic biochemical analyzer (Hitachi 7020, Japan). Lactate dehydrogenase (LDH) in the serum samples was determined by using LDH kit.

### Determination of tumor microvascular density after IV administration

Microvascular density (MVD) was checked by using the immuno – Bridge + kit. After the blood vessels of tumor tissue were dyed by immunohistochemical staining with PV-9000 and the endothelial cells were dyed brown, the blood vessels were yellowish-brown and could be easily recognized. First, under a microscope at low magnification, the most abundant areas of tumor angiogenesis, that is the ‘hot spots’, were selected and then the number of dyed brown capillaries was counted at 400 × magnification. The average of five horizons was determined as the MVD value. Brown stained endothelial cells, as long as they were separated from the adjacent capillaries, tumor cells, or other connective tissue, was considered to be a blood vessel. Although lumen often could be seen, these were not judged as blood vessels. In order to avoid the interference of larger blood vessels with the count, the blood vessels in which the tube wall was bundled around thick smooth muscle or with a lumen >8 red blood cells area, were not counted.

### Western blotting of VEGF and caspase 3 after IV drug treatment

A small piece of tumor tissue from each mouse was collected, grounded, cracked, and centrifuged. The protein concentrations of each diluted sample were determined by using BCA assay kit. The total protein (40 μg) and MAKER were resolved by electrophoresis and transferred to PVDF membranes. The conditions for membrane transfer were: VEGF, 200 mA, and 70 min; Caspase-3 and beta-actin, 200 mA, and 90 min. The membranes were blocked in TBST containing 5% skimmed milk for 2 hours at room temperature and then incubated with anti-caspase-3 antibody (1:800 dilution in TBST, Protein tech Group Inc, China) and anti-VEGF antibody (1:600 dilution in TBST, Bioworld, USA) overnight at 4 °C. After five full washes in TBST, the membranes were incubated with anti-mouse IgG antibody labeled with horseradish peroxidase (1:50000 dilution in TBST, Boster, China) for 2 hours. The membrane was exposed to X-ray film, which was rinsed, dried, and scanned, after which the gray value of films was computed by using BandScan software.

### Statistical analysis

All quantitative data are generated as mean ± standard deviation (S.D.). *t*-test was used to test the differences between groups. The significance level was set at a value of *p* < .05. *p* < .01 indicated the extremely significant difference.

## Results

### The characteristics of GAMCLCL

**Figure 1.**
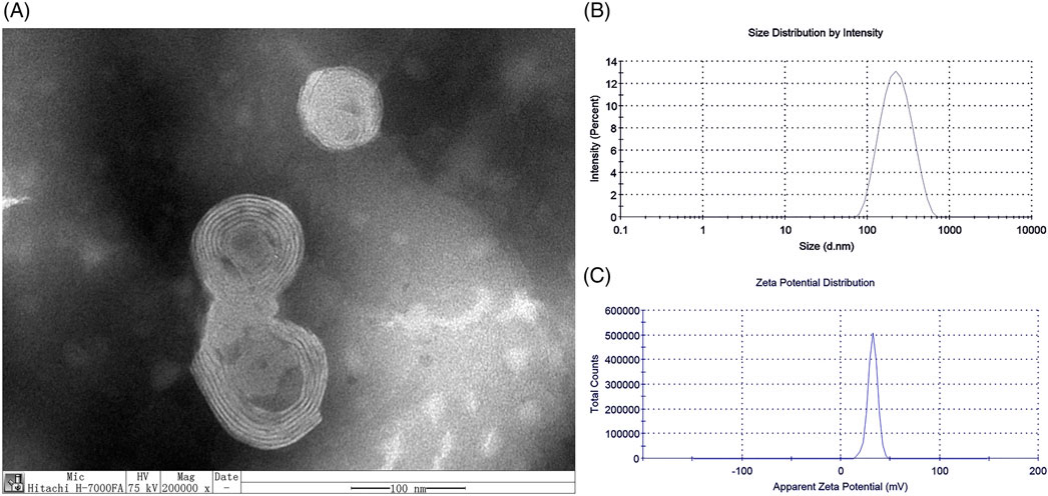
The physical characteristics of GAMCLCL. A: the morphology of GAMCLCL; B: the particle size of GAMCLCL; C: the potential of GAMCLCL.

GAMCLCL formed a clear, yellow, colloidal solution. The characteristics of GAMCLCL were shown in Figure 1. Transmission electron microscopy (Figure 1(A)) indicated that GAMCLCL had a regular spherical surface. The particle size was 194 ± 0.25 nm, PdI was 0.214 ± 0.02 (Figure 1(B)), and the potential was 31.9 ± 0.31 mv (Figure 1(C)). The mixture of GAMCLCL and serum did not show aggregation and precipitation within 72 hours, but the mixture of a control cationic liposome and serum resulted in immediate turbidity.

### GAMCLCL encapsulation efficiency

Curcumin is poorly soluble, with an EE of 98.26 ± 1.33%, and a DL was 3.93 ± 0.65%.

### Safety assay of GAMCLCL

**Figure 2.**
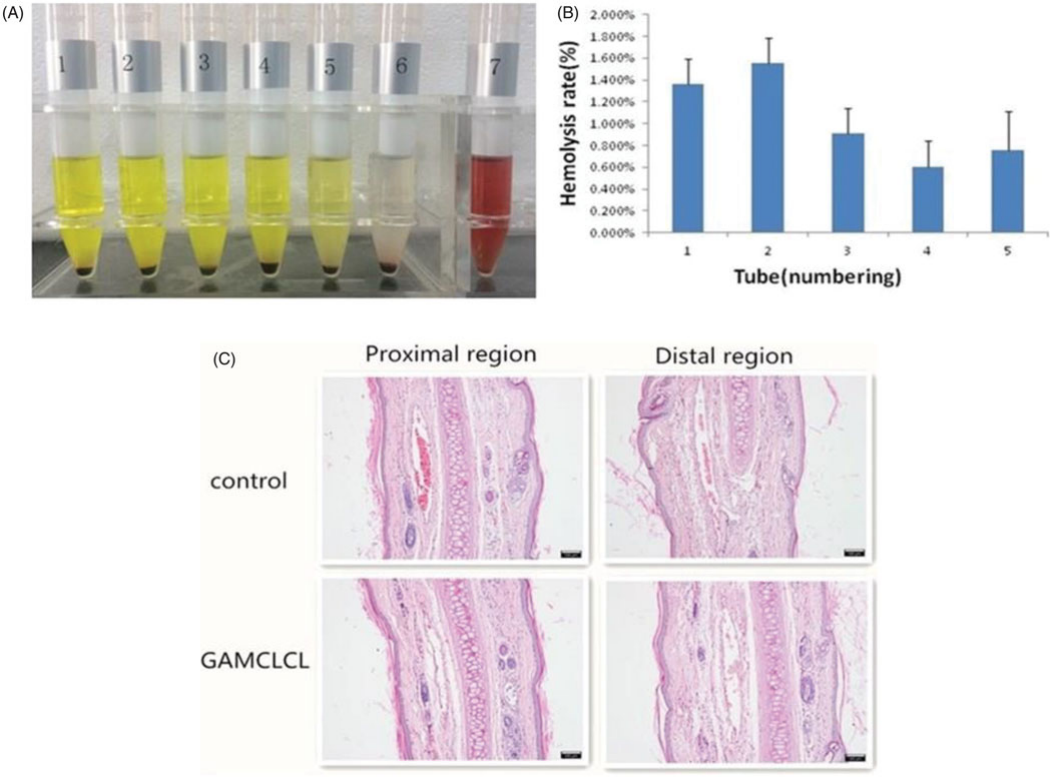
The safety results of GAMCLCL. A: Photograph of sample hemolysis test. Tube 1-5 are sample tube, their concentrations from high to low; tube 6 is negative tube; tube 7 is positive tube. B: the bar graph for hemolysis rate of GAMCLCL from tube 1 to 5. C: photograph of rabbit ear-rim vein slice of HE staining (100X). (The scale bar = 100 um).

The results of the hemolysis assays of GAMCLCL were shown in Figure 2. As shown in Figure 2(A), in the seventh tube (purified water), the clear red solution indicated a positive hemolytic result. The remaining 6 tubes did not form a clear red solution, and the red blood cells sank, after shaking, the red blood cells could be evenly dispersed. This represented negative hemolytic results. As shown in Figure 2(B), the hemolysis rate of tubes 1–5 was <5%. Generally, a hemolysis percentage of <5% is regarded as nontoxic and safe (Xiao et al., 2016). The hemolysis assay indicated that GAMCLCL had good hemocompatibility and was suitable for intravenous injection.

The pathological examination of rabbit vein was conducted and the results were shown in Figure 2(C). Compared with the control group, GAMCLCL resulted in no observable changes at the injection site, such as local swelling and inflammatory cell infiltration. No obvious pathological changes, such as thrombosis, necrosis, or hemorrhage, were identified by the HE staining of the rabbit vein. The injection of GAMCLCL into the rabbit marginal vein did not cause vascular irritation.

### The cellular test results *in vitro*

**Figure 3.**
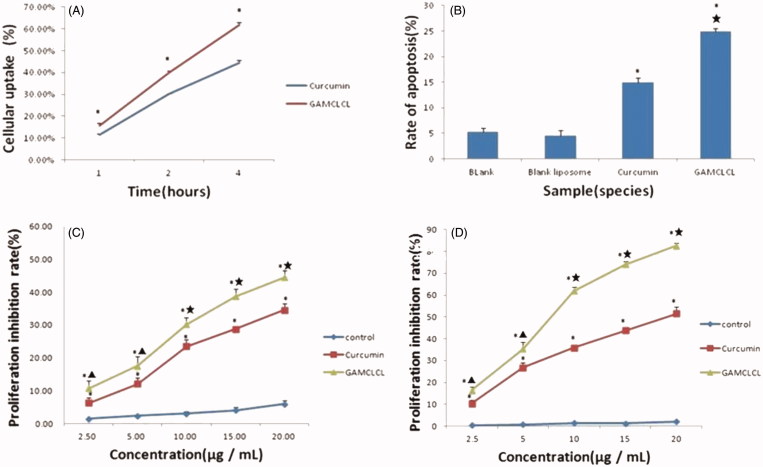
The results of cellular test *in vitro*. A: the line chart of cellular uptake by flow cytometry in 1, 2, 4 hours, respectively. Compared to free curcumin, *p < .01. B: the bar graph of cellular apoptosis rate by flow cytometry. Compared to two blank groups, *p < .01; compared to curcumin group, ★*p* < .01. C: the line chart of cytotoxicity at different concentrations within 24 hours. D: the line chart of cytotoxicity at different concentrations in 48 hours.

The cellular uptake of curcumin was shown in Figure 3(A). The results revealed that the uptake rate of GAMCLCL was much higher than that of free curcumin in 1, 2, and 4 hours (*p* < .01), respectively.

The results of cellular apoptosis were shown in Figure 3(B). Compared to the blank group, the apoptosis of HepG2 cells was significantly increased by treatment with GAMCLCL and free curcumin (*p* < .01); the cellular apoptosis induced by GAMCLCL was much stronger than that of free curcumin (*p* < .01).

Curcumin and GAMCLCL were added to HepG2 cells at various concentrations and incubated for 24 and 48 hours, respectively. Compared to curcumin-treated group, the cell proliferation inhibition rate in the GAMCLCL treated group was dramatically inhibited at 24 and 48 hours (Figure 3(C,D). However, the blank liposomes (the control) only exerted a slight inhibitory effect on HepG2 cell proliferation, which indicated that the blank liposomes induced hardly any cytotoxic effects.

### Antitumor efficacy of intratumoral administration

The antitumor efficacy of intratumoral administration was shown in Table 1. Compared to the tumor-bearing mice (model group), the tumor volume and tumor weight of mice in each treatment group were significantly reduced (*p* < .05 or *p* < .01). The tumor volume and tumor weight of three groups treated with GAMCLCL were negatively correlated with the dose. The tumor volume and tumor weight of the mice treated with high-dose GAMCLCL were not significantly different from the adriamycin-treated mice (both *p* > .05).

**Table 1.** Antitumor efficacy of intratumoral injection(IT) and intravenous injection(IV) (mean ± s) (*n* = 6).

Group	NBW(g)	Volume(mm^3^)	Tumor weight(g)	Inhibitory rate(%)
normal	29.15 ± 0.72			
model	24.32 ± 1.06*	1447.22 ± 187.90	3.96 ± 0.19	
Adriamycin				
IT	25.42 ± 0.93*	911.21 ± 104.01★	1.89 ± 0.12★	52.24
IV	27.96 ± 0.80△★	766.13 ± 87.96★	1.92 ± 0.19★	50.02
high dose				
IT	27.82 ± 0.57△★	937.54 ± 170.40★	2.18 ± 0.16★	45.01
IV	30.08 ± 0.53★	785.80 ± 143.94★	2.22 ± 0.19★	42.39
middle dose				
IT	26.99 ± 0.98△★	1179.90 ± 221.00★	2.61 ± 0.33★	33.97
IV	29.01 ± 1.15★	986.77 ± 183.48★	2.64 ± 0.33★	31.40
low dose				
IT	25.87 ± 0.78★	1213.48 ± 75.70★	3.00 ± 0.19★	24.33
IV	28.11 ± 0.86△★	1021.52 ± 64.08★	2.98 ± 0.28★	22.66
Curcumin				
IT	26.50 ± 0.78△★	1129.43 ± 241.30★	3.01 ± 0.12★	23.91
IV	28.69 ± 0.84★	1054.95 ± 106.71★	3.06 ± 0.14★	20.42

Adriamycin group: the dose of IT is 1 mg/kg, the dose of IV is 2 mg/kg; the other groups: the dose of IT and IV is the same. Compared to normal group, *p < .01, △*p* < .05; compared to model group, ★*p* < .01.

Adriamycin, a clinical antitumor agent, which was used as the positive control, caused a tumor inhibition rate of 52.24%. The high-dose GAMCLCL treated resulted in a tumor inhibition rate of 45.01%. Curcumin also caused antitumor effects, with a tumor inhibition rate of 23.91%, similar to that of the low dose of GAMCLCL (24.33%).

The net body weight (NBW) of mice was monitored as an index of systemic toxicity. Compared to the normal group, the NBW of all H22 tumor-bearing mice was significantly decreased (*p* < .01); the lowest NBW was found in the model group. Compared to the model group, weight gain was observed in all treatment groups (*p* < .05 or *p* < .01), and the NBW improvement of three GAMCLCL-treated groups were positively correlated with dose. The high- and medium-dose GAMCLCL treatment resulted in a higher NBW than adriamycin treatment (*p* < .01 and *p* < .05, respectively).

### Antitumor efficacy of intravenous administration

The antitumor efficacy of the intravenous administration was also shown in Table 1. Although the antitumor results of intravenous administration were very similar to the antitumor results of intratumoral administration, the antitumor efficacy of intravenous administration was slightly weaker than that of intratumoral administration, respectively.

### Blood biochemical analysis after IV administration

**Figure 4.**
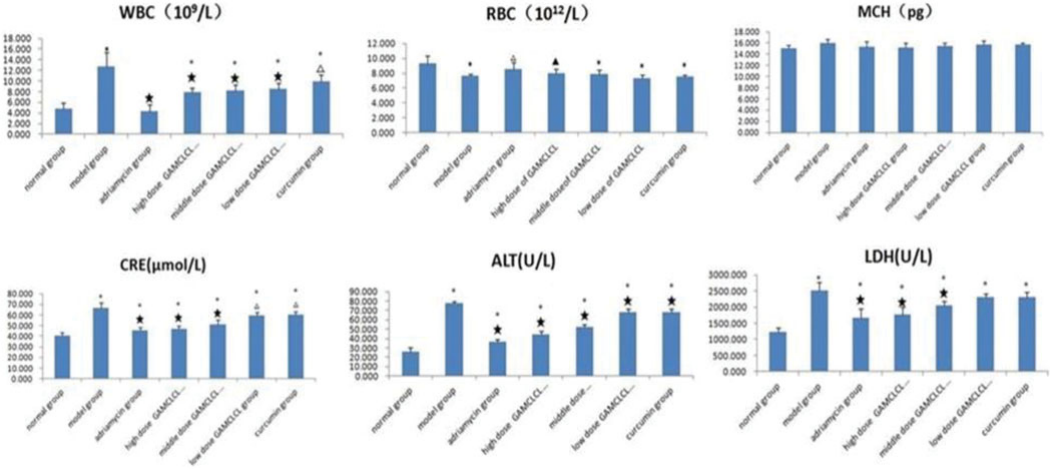
Bar graph of biochemical parameters (WRC, RBC, MCH, CRE, ALT, LDH). Compared to the normal group, ▲*p* < .05, *p < .01. Compared to the mode group, △*p* < .05, ★*p* < .01.

The results of blood biochemical tests were shown in Figure 4. Compared to the normal group, the RBC counts in the model group, the curcumin-treatment group and the three GAMCLCL-treatment groups was significantly decreased (*p* < .05 or *p* < .01), but the reduction observed in the adriamycin-treatment group (the positive control group) was not statistically significant (*p* > .05). The RBC counts of the three GAMCLCL-treatment groups were positively correlated to the treatment dose. Compared to the model group, the reduction in adriamycin-treated and the high dose GAMCLCL-treated group represented a significant amelioration of the decreased RBC count (*p* < .05, *p* < .01, respectively). The RBC count of the high dose GAMCLCL-treated group was similar to the adriamycin-treated group, with no statistical difference found between the two groups (*p* > .05).

In contrast, the WBC count was significantly increased in the model group, the curcumin group and the three GAMCLCL-treated groups in comparison with the normal group (*p* < .01). the WBC count was highest in the model group, and no statistically significant difference was observed between the adriamycin group and the normal group. The changes in the WBC count of the three GAMCLCL-treatment groups were negatively correlated with the GAMCLCL dose. Compared to the model group, all treatment groups cut down an increased WBC count, to varying degrees (*p* < .05 or *p* < .01).

The value of HGB was similar in all groups, with no statistical differences between the groups (*p* > .05).

ALT, CRE, and LDH are related to the function of liver, kidney, and heart, respectively. Compared to the normal group, the ALT, CRE, and LDH of all H22 bearing mice groups significantly increased (*p* < .01), the ALT, CRE, and LDH values in H22 tumor-bearing mice were significantly increased; the values were highest in the model group, which indicated that the functions of heart, liver, and kidney in H22 tumor-bearing mice were damaged. Nevertheless, the ALT, CRE, and LDH values in each treatment group were significantly reduced compared to the model group (*p* < .05 or *p* < .01). The ALT, CRE, and LDH values of the three GAMCLCL-treated groups were negatively correlated with the dose; furthermore, the reduction of CRE and LDH in the high dose GAMCLCL group was not significantly different from the adriamycin group, which indicated that GAMCLCL clearly improved the functions of heart, liver, and kidney of H22 tumor-bearing mice.

### MVD after IV drug treatment

**Figure 5.**
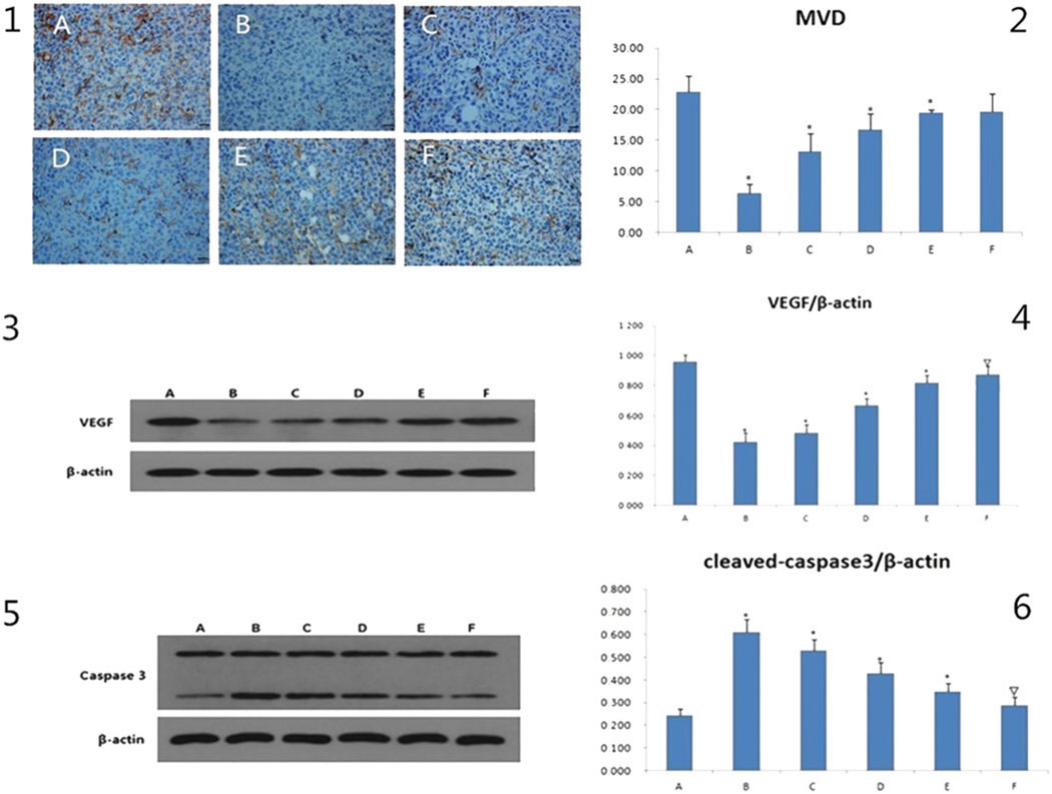
The results of MVD, VEGF, caspase3 *in vivo.* 1: the immunohistochemical pictures of MVD, the scale bar = 20 μm. 2: the bar graph of MVD of different groups, compared to mode group, *p < .01. 3 and 5 are the electrophoresis picture of VEGF and caspase3, respectively. 4 and 6 are bar graph of VEGF and caspase3, respectively. The β-actin is as internal standard, compared to mode group, △*p* < .05, *p < .01. A: mode group; B: adriamycin group; C: high dose of GAMCLCL group; D: middle dose of GAMCLCL group; E: Low dose of GAMCLCL group; F: curcumin group.

The immuno-histochemical pictures of MVD were shown in Figure 5(1), the brown regions indicated angiogenesis. The brown regions in the model group were markedly greater than in other groups. The MVD values were presented in a bar graph in Figure 5(2). Compared to the model group, the MVD of the curcumin group was not obviously different, but the values in the other drug administration groups were significantly decreased (*p* < .05 or *p* < .01). The MVD values of the three GANCLCL groups were negatively correlated with the dose.

### VEGF- and caspase 3- protein expression after IV administration

The western blots for the VEGF- and Caspase 3- proteins were shown in Figure 5(3,5), the intensity of the bands for VEGF and Caspase 3 indicated their expression levels.

The values of VEGF and Caspase 3 were presented in a bar graph in Figure 5(4 and 6), respectively. Compared to the model group, the expressions of VEGF were significantly decreased in each treatment group (*p* < .05 or *p* < .01). The effect of high dose of GAMCLCL was similar to the effect of adriamycin group. VEGF expression in the three GANCLCL groups was negatively correlated with the drug dose.

In contrast, compared to the model group, the expressions of Caspase 3 were significantly increased in each treatment group (*p* < .05 or *p* < .01). The Caspase 3 expression in the three GANCLCL groups was increased as the dose increased.

## Discussion

At present, chemotherapy is an important treatment for malignant tumors, the basic idea of chemotherapy is to treat liver cancer by using ‘toxic’ or ‘injury’ drugs (adriamycin, 5-FU, etc) to cause necrosis of liver cancer cells or tissues, however, its efficacy and clinical application are limited by its side effects. Some patients obtained temporary hepatic tumor shrinkage, their survival rate and survival time were not improved. In addition, the quality of life is also adversely affected. Therefore, in order to low the side effects, we attempt to adopt nontoxic curcumin as drug for the treatment of liver cancer.

As previously described, curcumin is an effective anti-cancer agent (Zhou et al., 2017), but its application has been hindered by its water insolubility, degradation at alkaline pH, low bioavailability. To overcome the barriers of curcumin in clinical setting application, several delivery systems such as liposomes, nano-particle, solid disperse (SD), and self-microemulsified (SME) curcumin (Liu et al., 2012) have been established. In this paper, we prepared a novel delivery system – glycyrrhetinic acid-modified curcumin-Loaded cationic liposomes (GAMCLCL), which could also overcome the limitations, and the tests for hemolysis and vascular irritation indicated that GAMCLCL had good hemocompatibility and low vascular irritation, and was suitable for intravenous injection.

However, curcumin is a kind of yellow pigment and spice of turmeric and curry, it is very safe, on the other hand, its anticancer activities are much lower than common chemotherapy drugs, so it is necessary to enhance the antitumor efficacy of curcumin. We modified cationic liposomes with glycyrrhetinic acid (GA), for the combination of curcumin and GA can improve the inhibition of the proliferation and promote the apoptosis of HepG2 cells (Mingxiang et al., 2017). In order to detect the new delivery system of GAMCLCL, we investigated the anticancer activities of curcumin and GAMCLCL *in vitro* and *in vivo*.

*In vitro*, compared to free curcumin, GAMCLCL displayed higher cellular uptake, promoted the apoptosis of HepG2 cells, and enhanced the inhibition of HepG2 cell proliferation. The *in vivo* antitumor activities of GAMCLCL were evaluated by intratumoral injection and intravenous injection. In the two administration experiments, the antitumor results of GAMCLCL after intravenous administration were very similar to those after intratumoral administration. In general, intratumoral injection has stronger anti-tumor effect than intravenous injection. In the present study, adriamycin was a positive control, its tumor inhibition rate of 1 mg/kg in intratumoral injection was 52.24%, and that of 2 mg/kg in intravenous injection was 50.02%, these indicated that intratumoral injection of adriamycin had stronger anti-tumor effect than intravenous injection. However, the antitumor results of the three-dose of GAMCLCL after intravenous administration were very similar to those after intratumoral administration, respectively, the results demonstrated indirectly that GAMCLCL had good targeting of tumor.

Changes in blood biochemical parameters can reveal pathologic changes in the physiological functions of the body. Compared to the normal group, the blood biochemical indicators of H22 bearing mice were significantly altered, the RBC count was decreased, the WBC count was increased (Wenrong Shi et al. also reported this result) (Shi et al., 2015); and the serum ALT, CRE, and LDH values were increased. Nevertheless, compared to the model group, the injection of GAMCLCL significantly improved the above blood biochemical indicators and simultaneously improved the function of the heart, liver, and kidney in H22 tumor-bearing mice.

The tumor inhibition rate in both administration routes indicated the definite antitumor effect of free curcumin at 20 mg/kg, which was similar to low-dose GAMCLCL (5 mg/kg), therefore, the antitumor effects of GAMCLCL were much stronger than that of free curcumin.

The change in body weight is an indicator of the systemic toxicity of a drug (Shaomei et al., 2016). Compared to the model group, the NBW of the three GAMCLCL-treated groups was greatly elevated; the NBW in the high- and middle-dose of GAMCLCL-treated groups was higher than that of the adriamycin group, which indicated that the systemic toxicity of GAMCLCL was lower than that of adriamycin.

Some studies have confirmed that the MVD is associated with the tumor growth rate and the survival term of patients (Zolota et al., 1999; Yang et al., 2015). In this paper, the experiment demonstrated that MVD values in the GAMCLCL-treated tumor-bearing mice were significantly lower than the model group (*p* < .01), which indicated that GAMCLCL enhanced the inhibitory effects of curcumin on tumor growth and metastasis.

VEGF is currently the strongest active and specific vascular growth factor, able to stimulate tumor vascular endothelial proliferation, migration, the induction of angiogenesis, and promotion of tumor growth and metastasis. HCC is considered as a hypervascular tumor, in which tumor growth and metastasis are angiogenesis-dependent (Young-Sam et al., 2002). Our data showed that the protein and mRNA expression of VEGF in the GAMCLCL-treated groups were significantly down-regulated in a dose-dependent manner compared to the model group, which indicated that GAMCLCL inhibited tumor vascular formation, tumor growth, and metastasis.

Caspase 3 is the main executor of the process of apoptosis and the most important effect factor. The activation of caspase-3 is an important mechanism of cell apoptosis; the inactivation or anomalies expression is related to the occurrence and development of a wide variety of tumors (Patrick et al., 2013). The results showed that GAMCLCL up-regulated the expression of caspases-3 protein and mRNA compared to the model group, which indicated that they increased the induction of tumor cell apoptosis.

It is believed that the anticancer and cancer prevention activities of curcumin occur through the modulation of several signaling pathways, cell cycle, transcription factors, growth factors, and their receptors, invasion, cytokines, enzymes, and the gene regulation of cell proliferation and apoptosis (Teiten et al., 2010; Basnet & Skalko-Basnet, 2011; Wilken et al., 2011; Huiqiang et al., 2017). In this study, we confirmed the anti-tumor activity of curcumin and GAMCLCL *in vitro* and *in vivo*, and the antitumor activities of GAMCLCL were much stronger than those of free curcumin. Curcumin and GAMCLCL exerted their anti-tumor activities by inhibiting of HepG2 cell proliferation, inducing of HepG2 cell apoptosis, reducing of microvascular formation, and inhibiting of tumor angiogenesis and metastasis by decreasing MVD values, down-regulating VEGF, up-regulating caspases 3.

Adriamycin is a common anticancer chemotherapeutical agent. The adult dosage is 40–60 mg/m^2^ and the total dose per body surface area should not exceed 400 mg/m^2^. In this study, adriamycin was used as a positive control. The dose administered to the mice (2 mg/kg/day for 7 days) was equivalent to a human dose of 445 mg/m^2^, as determined from the dose-transfer equation between mouse and human, therefore, the experimental results of adriamycin in this paper could serve as a practical reference. The effects of adriamycin in this paper on most of the studied parameters were stronger than those of GAMCLCL and free curcumin. However, the tumor inhibition rate of high dose GAMCLCL was only slightly weaker than that of adriamycin, the systemic toxicity of GAMCLCL was lower than that of adriamycin, therefore, the antitumor effects of GAMCLCL were worthy of further study.

In addition, cationic liposomes, a class of non-viral vectors, have usually been studied for the delivery of nucleic acid therapeutics (Yuan et al., 2011; Ojea-Jimenez et al., 2012). Despite recent progress, cationic liposomes still suffer from some limitations. Li et al. (1999) reported that the immediate effect of serum on cationic liposomes was aggregation. Moreover, cationic liposomes can easily combine with negatively charged components, such as plasma protein and red blood cells in the blood and lead to pulmonary embolism when applied *in vivo* (Makiya et al., 2005). Therefore, cationic liposomes are usually used *in vitro*. In this study, the potential of GAMCLCL was +31.9 mv, which proved their cationic nature, but when mixed with serum, no aggregation and precipitation occurred over 72 hours. After the daily intravenous injection of GAMCLCL in mice for 7 days, the mice grew well, displayed normal activities and breathing, and no deaths occurred. The experimental results revealed that cationic liposomes could be used to encapsulate the curcumin, and the modification of cationic liposome with GA might shade the positive charge, which could overcome the limitations of cationic liposomes, improve the stability of liposomes in serum and *in vivo*, and could be injected by IV.

## Conclusion

GAMCLCL, a novel cationic liposomes delivery system, was prepared by using ethanol injection method, which exhibited reasonable values of particle size, the potential, and EE%. Although GAMCLCL was a cationic liposome, could be Intravenously administrated by modified with GA. Compared to free curcumin, GAMCLCL exhibited much stronger antitumor activities *in vitro* and *in vivo*. The antitumor results of GAMCLCL after intravenous administration were very similar to those after intratumoral administration. Curcumin is a nontoxic natural compound, its antitumor effects are weaker than that of adriamycin, but can be improved by prepared of GAMCLCL. The experimental results indicated that GAMCLCL was a successful delivery system, that overcame the limitations of curcumin applying and cationic liposomes, and worthy of further study.
